# Supplementary material for: TORC2-Gad8-dependent myosin phosphorylation modulates regulation by calcium
Source: eLife. 2019 Sep 30;8:e51150. doi: 10.7554/eLife.51150 (PMC6802964; doi:10.7554/eLife.51150)
Supplement: Supplementary file 1. [file elife-51150-supp1.docx]

**Supplementary Table 1**: *Strains used during this study.*

**Strain Source**

wild type h^-^ Lab stock

*cdc10.v50* h^-^ (Nurse et al, 1976)

*cdc25.22* h^-^ (Nurse et al, 1976)

*myo1::kanMX6* h^-^ (Sirotkin *et al*, 2005)

*cam2::URA4 ura4.d18* h^-^ (Itadani *et al*, 2007)

*sla2.mCherry:natMX6* h^-^ (Alvarez-Tabarés *et al*, 2007)

*leu1::nmt81gfp.act1:URA4 ura4.d18* h^90^ (Doyle *et al*, 2009)

*LifeACT.mCherry:LEU2 leu1.32* h^-^ (Huang *et al*, 2012)

*acp1.gfp:kanMX6* h^-^ (Baker *et al*, 2016)

*ste20::kanMX6* h^-^ (Baker et al, 2016)

*gad8::kanMX6* h^-^ (Baker et al, 2016)

*myo1.S742A:URA4 ura4.d18* h^-^ This study

*myo1.S742D:URA4 ura4.d18* h^-^ This study

*mNeongreen.myo1:URA4 ura4.d18* h^-^ This study

*mNeongreen.myo1.S742A:URA4 ura4.d18* h^-^ This study

*mNeongreen.myo1:URA4 ura4.d18* h^90^ This study

*mNeongreen.myo1.S742A:URA4 ura4.d18* h^90^ This study

*mNeongreen.myo1:URA4 cam2::URA4 ura4.d18* h^-^ This study

*yfp.myo1:kanMX6 sid4.tdTomato:hphMX6* This study

*cam1.gfp:kanMX6* h^-^ This study

*cam1.gfp:kanMX6 myo1::kanMX6* h^-^ This study

*cam1.gfp:kanMX6 myo1.S742A:URA4 ura4.d18* h^-^ This study

*cam1.gfp:kanMX6* h^90^ This study

*cam1.gfp:kanMX6 myo1.S742A:URA4 ura4.d18* h^90^ This study

*cam2.gfp:kanMX6* h^-^ This study

*cam2.gfp:kanMX6 myo1::kanMX6* h^-^ This study

*cam2.gfp:kanMX6 myo1.S742A:URA4 ura4.d18* h^-^ This study

*cam1.gfp:kanMX6 cam2::URA4 ura4.d18* h^-^ This study

*cam2.gfp:kanMX6* h^90^ This study

*cam2.gfp:kanMX6 myo1.S742A:URA4 ura4.d18* h^90^ This study

*leu1::nmt41gfp.myo1:URA4 ura4.d18 cam1.mCherry:hphMX6* h^-^ This study

*cam1.mCherry:hphMX6 cam2.gfp:kanMX6* h^-^ This study

*sla2.mCherry:natMX6 myo1.S742A:URA4 ura4.d18* h^-^ This study

*LifeACT.mCherry:LEU2 leu1.32 myo1::kanMX6* h^-^ This study

*LifeACT.mCherry:LEU2 leu1.32* h^90^ This study

*myo1.S742A:URA4 ura4.d18 sla2.mCherry:natMX6* h^-^  This study

*leu1::nmt81gfp.act1:URA4 cam2::kanMX6 ura4.d18* h^90^ This study

*leu1::nmt81gfp.act1:URA4 myo1.S742A:URA4 ura4.d18* h^90^ This study

*mNeongreen.myo1:URA4 cam2::kanMX6 ura4.d18* h^90^ This study

*cam1.gfp:kanMX6 cam2::URA4 ura4.d18* h^90^ This study

**References**

Alvarez-Tabarés I, Grallert A, Ortiz J-M & Hagan IM (2007) Schizosaccharomyces pombe protein phosphatase 1 in mitosis, endocytosis and a partnership with Wsh3/Tea4 to control polarised growth. *J. Cell. Sci.* **120:** 3589–3601

Baker K, Kirkham S, Hálová L, Atkin J, Franz-Wachtel M, Cobley D, Krug K, Macek B, Mulvihill DP & Petersen J (2016) TOR complex 2 localises to the cytokinetic actomyosin ring and controls the fidelity of cytokinesis. *J. Cell. Sci.* **129:** 2613–2624

Doyle A, Martin-Garcia R, Coulton AT, Bagley S & Mulvihill DP (2009) Fission yeast Myo51 is a meiotic spindle pole body component with discrete roles during cell fusion and spore formation. *J. Cell. Sci.* **122:** 4330–4340

Huang J, Huang Y, Yu H, Subramanian D, Padmanabhan A, Thadani R, Tao Y, Tang X, Wedlich-Soldner R & Balasubramanian MK (2012) Nonmedially assembled F-actin cables incorporate into the actomyosin ring in fission yeast. *The Journal of Cell Biology* **199:** 831–847

Itadani A, Nakamura T & Shimoda C (2007) Localization of type I myosin and F-actin to the leading edge region of the forespore membrane in Schizosaccharomyces pombe. *Cell Struct. Funct.* **31:** 181–195

Nurse P, Thuriaux P & Nasmyth K (1976) Genetic control of the cell division cycle in the fission yeast Schizosaccharomyces pombe. *Mol. Gen. Genet.* **146:** 167–178

Sirotkin V, Beltzner CC, Marchand J-B & Pollard TD (2005) Interactions of WASp, myosin-I, and verprolin with Arp2/3 complex during actin patch assembly in fission yeast. *The Journal of Cell Biology* **170:** 637–648
