# Supplementary material for: TORC2-Gad8-dependent myosin phosphorylation modulates regulation by calcium
Source: eLife. 2019 Sep 30;8:e51150. doi: 10.7554/eLife.51150 (PMC6802964; doi:10.7554/eLife.51150)
Supplement: Supplementary file 2. [file elife-51150-supp2.docx]

**Supplementary Table 2**: *Oligonucleotides used during this study.*

**Olig # Name Sequence (5’-3’)**

226 5’Nde1cam1 CATATGACTACCCGTAACC

227 3’BamH1cam1 GGATCCCTACTTGGAAGAAATG

393 5’Nde1cam2 CATATGCCTGCCTCCAAAGAACAAACCG

394 3’BamH1cam2 GGATCCCTATTTTGCCATGATTCTCTG

403 5’Xho1YPet CTCGAGATGGTGAGCAAAGGCGAAGAGC

404 3’BamH1H6G3YPet GGATCCTTAatgatgatgatgatgatgaccaccaccCTTATAG

AGCTCGTTCATGCCC

405 5’Nde1CyPet CATATGGTGAGCAAGGGAGAGG

406 3’CyPetMyo1IQ1Xho1 CTCGAGAGCTTCAGATCTTCTTCTAACATAAGAACGCCAAGCA

CGTTGTATACGGGTTGCCATGTCGACTTTGTACAGTTCGTCCA

TGCCGTGGGTG

425 BglII Myo1IQ12 Xho1F GATCcGAAGCTgctgcttgtattcagaagttgtggaataggaa

caaagttaacatggaacttgaaC

426 BglII Myo1IQ12 Xho1R TCGAgttcaagttccatgttaactttgttcctattccacaactt

ctgaatacaagcagcAGCTTCg

427 IQ12 S742D F CTTATGTTAGACGTCGCGACGAAGCTGCTGCTTG

428 IQ12 S742D R CAAGCAGCAGCTTCGTCGCGACGTCTAACATAAG

429 Sal1Myo1IQ2BglIIF tcgacgctgctgcttgtattcagaagttgtggaataggaacaa

agttaacatggaacttgaactcgaga

430 Sal1Myo1IQ2BglIIR gatctctcgagttcaagttccatgttaactttgttcctattcca

caacttctgaatacaagcagcagcg

Note: Underscored sequences denotes endonuclease recognition sites
